# Patient-related barriers and enablers to the implementation of high-value physiotherapy for chronic pain: a systematic review

**DOI:** 10.1093/pm/pnad134

**Published:** 2023-09-28

**Authors:** Cameron Dickson, Rutger M J de Zoete, Carolyn Berryman, Philip Weinstein, Kexun Kenneth Chen, Paul Rothmore

**Affiliations:** School of Allied Health Science and Practice, The University of Adelaide, Adelaide, 5005, Australia; School of Allied Health Science and Practice, The University of Adelaide, Adelaide, 5005, Australia; Allied Health and Human Performance Unit, IIMPACT in Health, The University of South Australia, Adelaide, 5001, Australia; Hopwood Centre for Neurobiology, South Australian Health and Medical Research Institute, Adelaide, 5000, Australia; Brain Stimulation, Imaging and Cognition Group, The University of Adelaide, Adelaide, 5000, Australia; School of Public Health, The University of Adelaide, Adelaide, 5000, Australia; South Australian Museum, Adelaide, 5000, Australia; School of Allied Health Science and Practice, The University of Adelaide, Adelaide, 5005, Australia; School of Allied Health Science and Practice, The University of Adelaide, Adelaide, 5005, Australia

**Keywords:** physical therapy, chronic pain, osteoarthritis, low back pain, knee pain, neck pain, rheumatoid arthritis

## Abstract

**Objective:**

To identify and synthesize patient-related barriers to and enablers of the implementation of high-value physiotherapy (HVP) for chronic pain. Furthermore, to review what patient-related interventions have been used to facilitate the implementation of HVP for chronic pain, as well as their efficacy.

**Methods:**

We systematically searched the APA PsycInfo, Embase, CINAHL, Medline, Scopus, and PEDro databases for peer-reviewed studies (published in English) of adults with chronic pain. We used the Theoretical Domains Framework of behavior change to synthesize identified themes relating to barriers and enablers. Outcomes from studies reporting on interventions were also qualitatively synthesized.

**Results:**

Fourteen studies reported on barriers and enablers, 8 of which related to exercise adherence. Themes common to barriers and enablers included perceived efficacy of treatment, interrelationship with the physiotherapist, exercise burden, and the patient’s understanding of exercise benefits. Other barriers included fear of movement, fragmented care, and cost. Ten studies explored interventions, 9 of which aimed to improve exercise adherence. Of these, evidence from 4 randomized controlled trials of technology-based interventions demonstrated improved exercise adherence among intervention groups compared with controls.

**Conclusion:**

Patients with chronic pain experience barriers to HVP, including their beliefs, the nature of their interaction with their physiotherapist, perceived treatment efficacy, and cost. Enablers include rapport with their physiotherapist, achievable exercises, and seamless cost-effective care. Technology-based interventions have demonstrated effectiveness at increasing exercise adherence. Our findings suggest that interventions seeking to enhance implementation of HVP need to consider the multifactorial barriers experienced by patients with chronic pain.

**Study registration:**

Open Science Framework (https://doi.org/10.17605/OSF.IO/AYGZV).

## Introduction

Chronic pain is a major cause of disease burden across the globe.^1^ Chronic pain is pain that persists beyond 3 months,^2^ and it is associated with depression,^3^ reduced physical activity, disability,^4^ opioid-related harm,^5^ and suicidality.^6^ Individuals experiencing chronic pain are frequently managed by physiotherapists.^7^^,^^8^

Despite evidence supporting contemporary physiotherapy management for common chronic pain conditions, treatments lacking efficacy remain widely implemented.^9^^,^^10^ Examples include manual therapy, acupuncture, taping techniques, and the use of electrophysical agents.^11^^,^^12^ Persistent implementation of such treatments has given rise to the concept of “low-value” physiotherapy, which yields little or no benefit relative to cost, or where the risk of adverse impact is greater than the likelihood of benefit.^13^ Conversely, a recent consensus statement from Australian physiotherapists defined “high-value” physiotherapy (HVP) as “care that delivers most value for the patient, and the clinical benefits outweigh the costs to the individual or system providing the care”^14^ (p. 4). As a recent concept,^15^ HVP is often considered interchangeable with evidence-based practice (EBP). Although EBP is a key component of HVP, the latter has a clearer emphasis on cost-effectiveness and value.^16^ It is critically important to consider the influence of value, given the high percentage of physiotherapists who deliver care in the private sector (eg, 72% in Australia).^17^ Indeed, the value proposition of physiotherapy for patients and the broader primary health care system is a key component of a recent strategic plan released by the Australian Physiotherapy Association.^18^ Value proposition also underpins the international “Choosing Wisely” initiative, which aims to reduce the provision of low-value health care and is subscribed to by multiple national physiotherapy associations.^19^ The Choosing Wisely initiative aims to reduce the provision of low-value care.

In terms of HVP, active physiotherapeutic modalities are supported for the management of chronic pain conditions, including chronic low back pain (CLBP), chronic neck pain (CNP), and osteoarthritis (OA).^20–22^ Exercise therapy independently, and in combination with manipulative therapy and pain neuroscience education, has demonstrated efficacy for CLBP.^20^ As part of HVP, exercise is also supported for CNP, with moderate effects from strengthening exercises (ie, of the upper quadrant and neck regions) and smaller effects from other forms of exercise (eg, motor control exercises of the head–neck).^23^ For OA, HVP supports rehabilitation comprising aerobic and resistance-based exercise, weight management where indicated, and self-management.^22^

Patients’ perception of value (contributing to their engagement) is pivotal to the success of active and high-value treatment approaches and presents a substantial challenge to the clinical implementation of these approaches.^24^^,^^25^ Endeavors to improve the implementation of HVP have explored physiotherapist-related barriers and enablers and tested interventions that have focused on changing the decision-making and clinical behaviors of physiotherapists.^26–28^ However, factors including the patients’ perceptions of their condition, treatment expectations, health literacy, and misinformation can also influence the clinical management provided.^29–32^ The emergence of consumer-driven health care^33^ also highlights the importance of understanding patient-related influences on HVP implementation.

### Objectives

This systematic review aims to identify, evaluate, and summarize the patient-related barriers to and enablers of the success of implementing HVP for chronic pain. Furthermore, we aimed to describe the evidence for the efficacy of patient-related interventions that have been used to facilitate the implementation of HVP for chronic pain.

## Methods

As HVP and EBP are similar concepts and have been used interchangeably in the literature, we sought investigations incorporating either. To be eligible for inclusion in this review, studies were required to investigate patient-related barriers to or enablers of the implementation of HVP (or EBP) chronic pain management (review question 1) or interventions aimed at enhancing implementation of HVP (or EBP) for chronic pain (review question 2). Barriers and enablers were considered patient related if they were directly reported by patients; interventions were considered patient related if the focus was to directly engage patients in behavior change. For inclusion, studies were required to be peer reviewed, to be published in English, and to pertain to adult persons 18 years of age or more. The completed eligibility criteria are summarized in Table 1.

**Table 1.** Inclusion and exclusion criteria applied to publications from systematic search.

Inclusion criteria	
1	The full article is available for review.
2	The article is published in English.
3	The article focuses on physiotherapy as the health care provider.
4	The study involves adults (ie, ≥18 years of age).
5	The article reports on chronic (>3 months’ duration) musculoskeletal pain as the health condition of interest.
6	The article is a peer-reviewed publication.
7	The article reports on patient-related barriers to or enablers of the implementation of high-value HVP or EBP chronic pain management.^a^
8	The article reports on an intervention to enhance implementation of HVP or EBP chronic pain management.^a^
**Exclusion criteria**	
1	The article pertains to non-neuromusculoskeletal chronic pain (eg, cancer pain, migraine, neuropathic pain).
2	The article pertains to non-physiotherapeutic management (eg, osteopathy, chiropractic).
3	The full article does not report on analyses of primary data (eg, is an editorial, letter to the editor, short communication, or study protocol).
4	The full article is a review of literature.
5	The article is a thesis.
6	The study involves healthy participants.

*Abbreviations:* EBP= evidence-based practice; HVP= high-value physiotherapy.

a Inclusion criteria 1–6 were applied to all publications during the screening process. Inclusion criteria 7 and 8 were applied (to publications meeting criteria 1–6) for the purposes of addressing questions 1 and 2, respectively, of the systematic review.

A systematic search was conducted within the following databases: APA PsycInfo, Embase, Cumulative Index to Nursing and Allied Health Literature (CINAHL), Medline, Scopus, and PEDro. The search strategy was devised through the use of a logic grid comprising key concepts, related synonyms, and index terms for each database, where required. Key concepts and search terms for each database are detailed in Supplemental Table S1. The search strategy was reviewed by an experienced Medical Librarian and checked against the Peer Review of Electronic Search Strategies checklist.^34^ The search was completed for titles, keywords, and abstracts on January 25, 2023. As an exemplar, the full search syntax for the Medline database is demonstrated in Supplemental Table S2.

**Figure 1.**
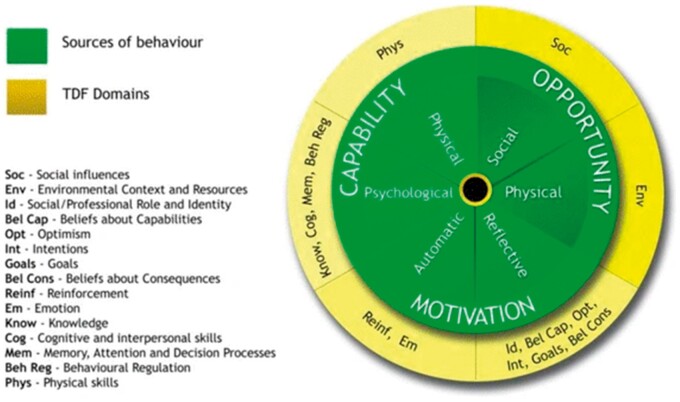
The articulating domains of the COM-B model and TDF.^37^ This figure demonstrates the domains of the Capability Opportunity Motivation—Behavior (COM-B) model (inner circle), as it articulates with the Theoretical Domains Framework of Behavior (TDF) on the outer circle. Via an a priori framework synthesis approach, the COM-B and TDF were used to synthesize themes arising from the qualitative studies included in the review.

This systematic review was guided by the Preferred Reporting Items for Systematic reviews and Meta-Analyses guidelines^35^ and used a framework synthesis approach.^36^ The Theoretical Domains Framework (TDF), interlinked with the Capability Opportunity Motivation—Behavior (COM-B) model (Figure 1), provided a robust a priori framework for synthesis of our data and reflects the multifactorial nature of HVP.^37^ The validated TDF and COM-B model have been widely used in implementation science research, including for the mapping of barriers and enablers influencing the implementation of health care guidelines both for clinicians and patients.^37–40^

### Protocol and registration

Consistent with PRISMA requirements, the protocol for this systematic review was registered and made publicly available via Open Science Framework (https://doi.org/10.17605/OSF.IO/AYGZV).

### Study selection

**Figure 2.**
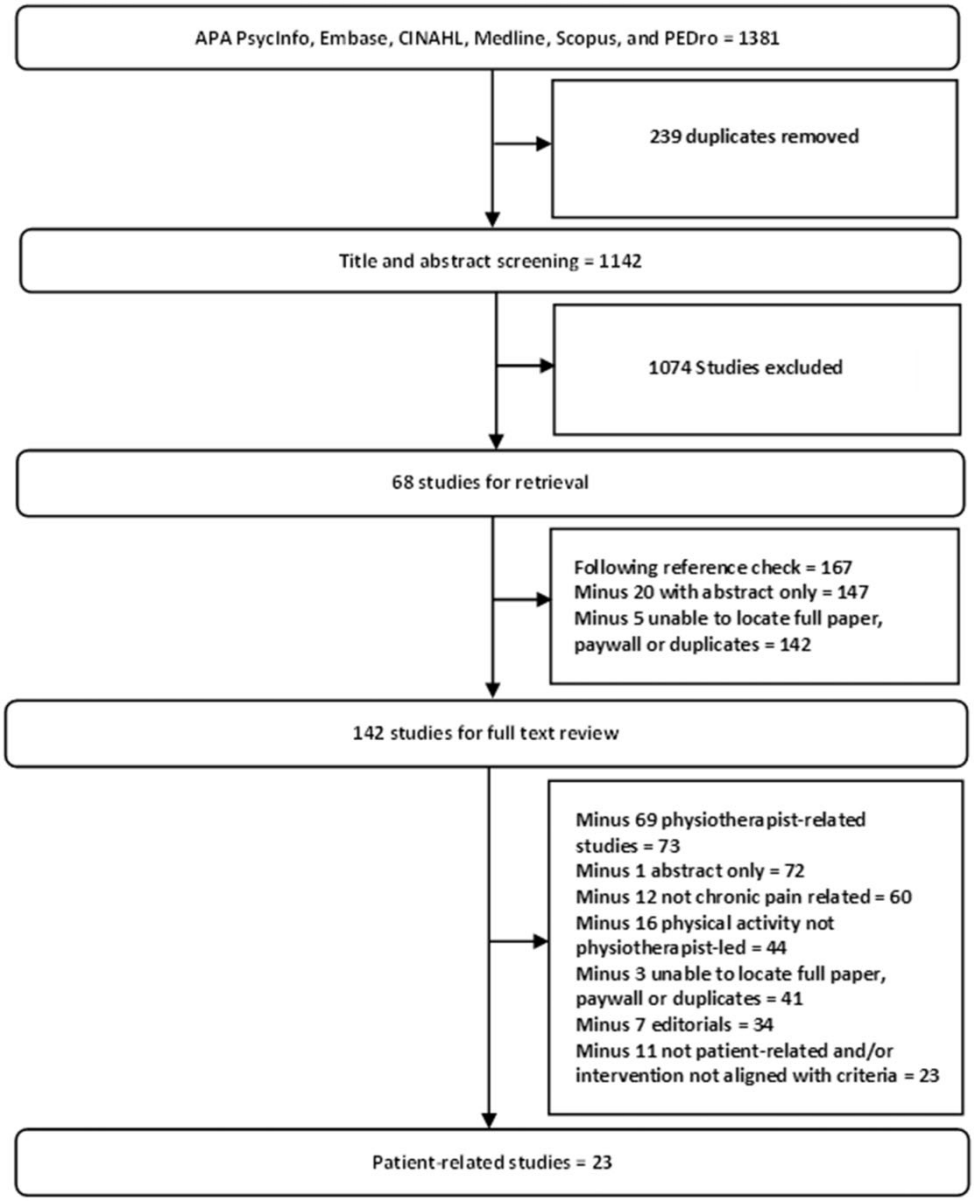
Flow chart representing the process of selecting the included studies. This figure demonstrates the study selection process completed for this review after systematic search of the APA PsycInfo, Embase, Cumulative Index to Nursing and Allied Health Literature (CINAHL), Medline, Scopus, and PEDro databases. The central text boxes in the figure denote each stage of the study selection process and the numbers of studies carried forward to each subsequent stage. The adjacent text boxes show the number of studies excluded at each stage and the reasons for doing so.

The study selection process is summarized in Figure 2. Results were downloaded into the Endnote X9 reference management software (Clarivate Analytics, Philadelphia, PA, United States) and transferred into the online platform Covidence (Veritas Health Innovation, Melbourne, Australia) with duplicates removed. Title and abstract screening was completed by 2 independent reviewers (C.D. and K.K.C.). A reference list search for included articles was completed by C.D. Full-text screening was completed by authors C.D. and K.K.C. Studies in which exercise and activity levels were the focus of treatment (eg, enhancing activity levels among patients or enhancing adherence to exercise) were included only if the program was led by a physiotherapist. Studies in which the sample of individuals comprised those with acute and chronic pain (and results were not reported separately) were excluded. Studies exploring feasibility / proof-of-concept of interventions were included.

### Risk-of-bias assessment

All of the included qualitative studies^24^^,^^41–53^ and randomized controlled trials^54–61^ were independently assessed for risk of bias by authors C.D. and P.R. Qualitative studies were assessed with the Joanna Briggs Institute Checklist for Qualitative Research (Supplemental Table S3),^62^ and the randomized controlled trials were assessed with the Joanna Briggs Institute Critical Appraisal Checklist for Randomized Controlled Trials (Supplemental Table S4).^63^ Disagreements between C.D. and P.R. were discussed until consensus was reached.

### Synthesis of studies

For the studies reporting on barriers and enablers, extraction of data themes and allocation to a domain of the COM-B/TDF was completed by one author (C.D.) and cross-checked by a second author (P.R.). Disagreements were discussed until consensus was reached. Guidelines for use of the TDF^37^ informed the identification and charting of themes, which was completed in the computer program Excel (Microsoft Corporation, Redmond, WA, United States). Overlapping narratives within studies (eg, where patients had reported multiple barriers to or facilitators of the implementation of HVP that were thematically consistent) were merged into common themes and qualitatively synthesized. Outcomes from the studies reporting on interventions were also qualitatively synthesized.

## Results

Our search and subsequent screening yielded 23 studies.^24^^,^^41–61^^,^^64^ Summary descriptive data are provided in Table 2 (studies of barriers and enablers) and Table 3 (studies of interventions). Of the 23 studies, 14 reported on barriers to and enablers of the implementation of EBP,^24^^,^^41–48^^,^^50–53^^,^^64^ and 10 reported on interventions aimed at enhancing the implementation of EBP for chronic pain.^45,49^^,^^54–61^ One study reported on both.^45^ Across all studies, a range of patients experiencing chronic pain was represented, including patients with hip OA (*n *=* *3), knee OA (*n *=* *14), patellofemoral pain (*n *=* *1), CLBP (*n *=* *5), rheumatoid arthritis (RA) (*n *=* *1), and CNP (*n *=* *1). Three studies included patients with various (nonspecified) musculoskeletal complaints (Tables 2 and 3).

**Table 2.** Included studies reporting on barriers to and enablers of high-value physiotherapy.

Author	Year	Origin	Aims	CP condition	Population and sample size	Methodology
Boyle et al.	2022	AU	Barriers to and enablers of referral practices	CLBP	17 individuals: 8 male, 9 female	Qual. (semi-structured interviews)
Joyce et al.	2022	USA	Explore patient experiences of physiotherapy	CLBP	12 individuals: 1 male, 11 female	Qual. (semi-structured interviews)
Teo et al.	2021	AU	Explore patients experiences of physiotherapy	Knee OA	24 individuals: 6 male, 18 female	Qual. (semi-structured interviews)
Garrett et al.	2021	USA	Barriers to and enablers of nonpharmacological treatment of CP	CP (various)	25 individuals: 9 male, 16 female	Qual. (semi-structured interviews)
Meerhoff et al.	2021	NL	Barriers to and enablers of PROMs	MSK (various)	21 individuals: 6 male, 15 female	Qual. (semi-structured interviews)
Smith et al.	2019	UK	Barriers to and enablers of exercise adherence	PFJ	20 individuals: 10 patients, 10 PTs	Qual. (interviews embedded in RCT)
Nicolson et al.	2018	AU and NZ	Barriers to and enablers of exercise adherence	Knee OA	373 individuals: 230 patients, 143 PTs	Quant. (survey)
Saner et al.	2018	Switzerland	Barriers to and enablers of exercise adherence	CLBP	44 individuals, 25 male, 19 female	Qual. (survey)
Danbjorg et al.^a^	2018	Denmark	Barriers to and enablers of exercise adherence	Hip and knee OA	6 individuals: 2 male, 4 female	Qual. (focus groups and workshops)
Palazzo et al.	2016	France	Barriers to and enablers of exercise adherence	CLBP	29 individuals: 12 male, 17 female	Qual. (semi-structured interviews)
Withall et al.	2016	UK	Barriers to and enablers of exercise (structured in-person programs)	RA	19 individuals: 4 male, 15 female	Qual. (focus groups)
Escolar-Reina et al.	2010	Spain	Barriers to and enablers of exercise adherence	CNP and CLBP	34 individuals: 11 male, 23 female	Qual. (focus groups)
Poitras et al.	2010	France	Barriers to and enablers of management recommendations	Knee OA	28 individuals: 11 patients, 7 GPs, 10 PTs	Qual. (focus groups)
Campbell et al.	2001	UK	Barriers to and enablers of exercise adherence	Knee OA	20 individuals: 6 male, 14 female	Qual. (interviews embedded in RCT)

*Abbreviations:* AU= Australia; CLBP= chronic low back pain; CNP= chronic neck pain; CP= chronic pain; GP= general practitioner; MSK= musculoskeletal; NL= Netherlands; NZ= New Zealand; OA= osteoarthritis; PFJ= patellofemoral joint; PROM= patient-reported outcome measure; PT= physiotherapist; Qual= qualitative; RA= rheumatoid arthritis; RCT= randomized controlled trial; UK= United Kingdom; USA= United States of America.

a Reports on barriers, enablers, and interventions and is present in Tables 1 and 2.

**Table 3.** Included studies reporting on interventions to enhance implementation of high-value physiotherapy.

Author	Year	Origin	Aims	CP condition	Population and sample size	Methodology	Intervention
Alasfour et al.	2022	SA	Improve exercise adherence	Knee OA	40 female (≥50 years of age): 20 control, 20 intervention	RCT	Smartphone application
Bennell et al.	2020	AU	Improve exercise adherence	Knee OA	110 individuals (50 years of age / BMI > 30): 56 intervention, 54 control	RCT	24-week SMS intervention
Osteras et al.	2019	Norway	Improve implementation of EBP	Hip and knee OA	40 GPs, 37 PTs; 393 individuals: 284 intervention, 109 controls	Cluster-RCT	Structured OA care model
Danbjorg et al.^a^	2018	Denmark	Improve exercise adherence	Hip and knee OA	6 individuals: 2 male, 4 female	Qualitative	Smartphone application
Li et al.	2018	Canada	Improve exercise adherence	Knee OA	60 individuals: 30 immediate, 30 delayed	Proof-of-concept RCT	Telephone coaching^b^
Bennell et al.	2017	AU	Improve exercise adherence	Knee OA	168 individuals (≥50 years of age): 84 intervention, 84 control	RCT	Telephone coaching
Lambert et al.	2017	AU	Improve exercise adherence	Upper and lower limb MSK	80 individuals: 40 intervention, 40 control	RCT	Online application^c^
Li et al.	2017	Canada	Improve exercise adherence	Knee OA	34 individuals (intervention): 6 male, 24 female	Feasibility RCT	Telephone coaching^b^
Hinman et al.	2016	AU	Improve exercise adherence	Knee OA	10 PT, 4 telephone coaches, 6 patients	Qualitative	Telephone coaching
Dar et al.	2014	Israel	Improve exercise adherence	Knee OA	14 individuals: 5 intervention, 9 controls	Pilot RCT	MMS

*Abbreviations:* AU= Australia; BMI= body mass index; CP= chronic pain; EBP= evidence-based practice; GP= general practitioner; MSK= musculoskeletal; MMS= multimedia messaging service; OA= osteoarthritis; PT= physiotherapist; RCT= randomized controlled trial; SA= Saudi Arabia; SMS= short message service.

a Reports on barriers, enablers and interventions and is present in Tables 1 and 2.

b Technology-enabled telephone-based coaching utilizing a “Fitbit Flex” wearable activity tracker.

c Application comprising option for use on smartphone and remote user support.

### Studies reporting on barriers and enablers

Of the 14 studies that investigated barriers to and enablers of HVP, alignment was found among 10 domains of the TDF and themes considered as barriers. Themes considered enablers aligned with 9 TDF domains. In total, 55 individual themes were identified as barriers and 40 as enablers.

#### Domains of the TDF that aligned with themes considered as barriers

***1. Knowledge.*** One study among patients with knee OA^48^ found that a lack of understanding of the benefits of exercise and uncertainty about what type and intensity of exercise to do were barriers to adherence. Patients also expressed uncertainty about how weight loss could be helpful for their condition. In patients with patellofemoral pain,^50^ some understood that their chronic pain symptoms related to tissue damage, reflecting a biomedical understanding of their pain. Biomedically oriented beliefs about chronic pain have been associated with reduced treatment adherence and fear-avoidant behaviors.^65^

***2. Memory, attention, and decision processes.*** One study of patients with CLBP reported that forgetfulness was a barrier to exercise adherence.^52^ Patients with hip and knee OA also reported difficulty remembering to do their exercises when not supervised.^45^ Among patients with various chronic pain conditions,^44^ one patient reported “…I can only absorb one thing … Physical therapy for me would work if they taught one thing” (p. 21).

***3. Beliefs about capabilities.*** Barriers to exercise adherence reported by patients with knee OA^24^ included “the willingness and ability to accommodate the exercises into everyday life” (p. 134). One study among patients with back and neck pain reported that insufficient feedback from physiotherapists on exercise performance was a barrier, contributing to a lack of confidence to continue and fear of doing exercises incorrectly.^47^ Another study of patients with CLBP found that for older persons or those not accustomed to regular physical activity, the perceived burden of exercise (distinct from the time taken to do the exercises) is a barrier to adherence.^46^ One patient reported that more than 3 or 4 exercises was too much for them to complete.

***4. Optimism.*** In patients with knee OA,^24^ prior health care experiences negatively influenced exercise adherence, including whether previous advice had provided a fatalistic prognosis. With regard to recommended weight loss, patients with knee OA^48^ felt this was difficult because of challenges undertaking physical activity and nonmodifiable predisposing factors, such as genetic influences. With regard to resumption or maintenance of work, patients were concerned about potential symptom exacerbation. With regard to structured in-person exercise, patients with RA^51^ were pessimistic about committing to programs of 6 weeks or longer, eg, “Twice a week for 6 weeks that’s a lot …” (p. 267).

***5. Intentions.*** Two studies involving patients with CLBP^52^ and hip and knee OA^45^ reported that lack of motivation to engage in exercise was a barrier.

***6. Beliefs about consequences.*** Perceived severity was a barrier to exercise adherence among individuals with knee OA.^24^ Conversely, one patient reported that they had “… got nothing to complain about” (p. 135), which also attenuated exercise compliance. Patients were less likely to adhere to exercise if they thought that their condition was caused by nonmodifiable factors, such as age or degenerative changes. Perceived efficacy also influenced exercise adherence: If the treatment was perceived as helpful, adherence was increased, and vice versa. This was a recurring theme in 3 studies involving patients with CLBP^46^^,^^47^^,^^52^ and CNP.^47^ With regard to patient-reported outcome measures (PROMs), patients thought some aspects were irrelevant, and the prospect of this information being shared with third parties such as insurers was a concern.^53^ One study of patients with CLBP^46^ found that unhelpful or false beliefs (underpinning a fear of movement) were a barrier to exercise. Exercise complexity was a barrier for some patients, relating to fear of hurting themselves when unsupervised. This theme also emerged in patients with hip and knee OA and RA.^45^^,^^48^^,^^51^ With regard to recommendations to resume or continue exercise, patients with knee OA recognized the importance of this but lacked an understanding of what benefits are associated with it.^48^ Patients with RA reported a preference for exercise programs to commence 6 months after diagnosis to ensure their symptoms were well managed by a stable medication regimen.^51^ Some patients with patellofemoral pain described an expectation that they would receive hands-on treatment, rather than self-managed exercises.^50^ Patient expectations (in terms of the treatment modality and effects) are known to influence results for persons with chronic pain,^66^ and the need for clinicians to navigate patient expectations has been recognized.^66^^,^^67^

***7. Reinforcement.*** Pain subsequent to exercise was a barrier to adherence among patients experiencing CLBP^48^ and knee OA.^46^ Patients with knee OA expressed frustration that their medication regimen did not provide sufficient pain relief to allow adequate physical activity levels.^48^

***8. Emotion.*** One study of patients with CLBP^46^ found that they experienced exercise programs as boring and repetitive. Despondency subsequent to longstanding symptoms, depressive symptoms, and lack of motivation were also considered barriers to adherence.^46^ Furthermore, transitioning from a supervised to a home-based exercise program resulted in feelings of abandonment from reduced follow-up and communication with clinicians.^46^

***9. Social influences.*** A lack of social support and perceived stigma associated with their condition were barriers to exercise adherence among patients with CLBP.^46^ Platforms such as social networks and forums to enhance adherence were explored in this study, which for some patients were considered a barrier because of confidentiality concerns.^46^ Another study of patients with CLBP^41^ reported that fragmented care, including insufficient interprofessional communication, was perceived as a barrier to care. Patients described a lack of formal referral between their general practitioner (GP) and physiotherapist, which for some was only done when prompted by the patient.^41^ Patients also described feelings of anger when no further options or onward referrals were made available to them if they were unresponsive to treatment. Lastly, patients described lack of trust in their physiotherapist as a barrier to reaching agreement on treatment options.^41^ One patient reported: “… I felt like he was doubting me the whole time and he didn’t believe that I was truly injured” (p. 7).^41^ Another study of patients with CLBP^42^ reported that a lack of connection between patient and physiotherapist was a barrier, with one patient stating, “The person that I had wasn’t really giving me their full attention ‘cause she was off doing other things, making phone calls” (p. 6).^41^ One study of patients with knee OA^48^ reported that physiotherapists prescribed exercises didactically, in contrast to recommendations for shared decision-making with patients.^68^ Patients with RA from one study^51^ indicated that exercising in groups (ie, the social context) was a deterrent. One patient reported that: “… all we did was sat around and talked about what was wrong with us” (p. 268).^51^

***10. Environmental context and resources.*** A study of patients with CNP and CLBP^47^ reported that exercise adherence was hampered if programs took too much time to complete. This theme also emerged from patients with knee OA^24^ and CLBP.^46^^,^^52^ Some patients also thought that using PROMs reduced treatment time.^53^ Lack of access to physiotherapy services and cost were barriers arising from 3 studies of patients with various chronic pain (various conditions), CLBP, and knee OA, respectively.^41^^,^^43^^,^^44^ In one study, one patient reported, “They only allowed you a total of six visits of physical therapy … Without the coaching I just can’t make myself do it alone” (p. 21).^44^ Similarly, patients with knee OA often stop attending physiotherapy because of cost.^43^

#### Domains of the TDF that aligned with themes considered as enablers

***1. Knowledge.*** Patients with CLBP and CNP^47^^,^^52^ reported that a clear explanation of their condition and treatment rationale was an important motivator of exercise adherence. One study of patients with patellofemoral pain reported that patients expected answers about what was causing their pain.^50^ Lastly, with regard to exercise programs, patients with RA valued the inclusion of an educational component on medications and how to manage symptoms of pain and fatigue.^51^

***2. Behavioral regulation.*** Patients with knee OA from one study^64^ valued a specific exercise plan with explicit parameters and with clear goals related to their function and symptoms.

***3. Beliefs about capabilities.*** The ability of patients to integrate exercise into day-to-day life was (if positive) considered an enabler of adherence among patients with both knee OA and patellofemoral pain.^24^^,^^50^ Among those with patellofemoral pain, a greater internal locus of control was important for exercise adherence, which was fostered by the provision of a manageable single-exercise program.^50^ Patients with CLBP^42^ reported feeling empowered by a combination of exercise and education.

***4. Optimism.*** Patients with CLBP^52^ valued the positive influence of exercises on functional activities such as walking and standing. Patients with RA^51^ reported that learning of others’ positive experiences of a structured in-person exercise program could promote adherence.

***5. Goals.*** With regard to structured in-person exercise programs, patients with RA^51^ reported that patient-centered goal setting was important so as to avoid “taxing goals being imposed by professionals and peer pressure” (p. 269). The importance of tracking individual progress and goal setting was also echoed by patients with hip and knee OA, for whom competition with oneself served as a motivator.^45^

***6. Beliefs about consequences.*** Patients experiencing severe pain or functional impairment from knee OA were more likely to have greater adherence to exercise.^24^ This related to the perceived efficacy of exercises, which was also an important influence on adherence among individuals with CNP and CLBP.^47^ Two studies of patients with CLBP also identified that exercise efficacy (ie, symptom management, functional gains, or as a preferred treatment over others) was an important influence on adherence.^46^^,^^52^ Patients with hip and knee OA were motivated to complete exercises if it was likely to prevent the need for surgical intervention.^45^ With regard to engagement with physiotherapy generally, perceived efficacy was an important factor reported in one study of individuals with various chronic pain conditions.^44^ Among patients with patellofemoral pain,^50^ treatment expectations reportedly influenced exercise adherence (ie, greater adherence if patients expected exercise as part of their treatment). With regard to PROMs, one study^53^ reported that patients viewed them as worthwhile and thought they aided the physiotherapist with assessment and diagnosis. Although some patients expressed concern about their PROM data being shared with third parties, others thought this might have some merit from a quality improvement perspective.

***7. Reinforcement.*** One study involving patients with CLBP^46^ found that symptom relief after exercise was an enabler of adherence. Furthermore, patients reported that exchange tools such as social networks and forums were likely to increase exercise adherence, if led by a professional who could answer questions.^46^ Patients reported that gaming technology was likely to enable exercise adherence, particularly if feedback on performance was provided.^46^

***8. Social influences.*** With regard to PROMs,^53^ patients found that they enabled communication with the physiotherapist, ie, if they were new patients or returning patients with a new problem. One study of patients with knee OA^64^ reported 3 themes that enabled exercise adherence, including review of progress with the physiotherapist with regard to pain, function, and exercise technique, as well as follow-up consults more than 3 months after the initial session to check on progress. Patients with knee OA also recognized the importance of communicating their needs and expectations to the physiotherapist,^48^ and they valued personalized care.^43^ Rapport and trust in the physiotherapist were also themes among patients with CLBP^42^ and knee OA.^43^ One patient with CLBP^42^ reported, “I was able to talk to them openly … I was able to just let them know when I was in pain. They didn’t judge me” (p. 6). Patients with RA^51^ valued exercising in a safe and supportive environment with social support and also found that telephone-based support fostered motivation and engagement. This was also the case for patients with hip and knee OA.^45^ One study among patients with CLBP^41^ reported that timely referral (be it to a physiotherapist from a GP, or onward referral to a different physiotherapist if not progressing) was an enabler of more effective management of their condition.

***9. Environmental context and resources.*** Patients with CLBP reported that keeping the exercises simple (and with no additional equipment) was important for ease of implementation.^52^ Engaging in a structured exercise program also enabled adherence for some of these patients. Patients with RA reported that accessibility of location was important for adherence to a structured in-person exercise program.^51^

### Studies reporting on interventions

Ten studies reported on interventions aimed at enhancing implementation of HVP, 9 of which aimed to improve exercise adherence (Table 3). Four of these studies used telephone-based coaching,^49^^,^^57^^,^^59^^,^^60^ 2 of which were technology enabled (ie, included the use of an activity tracker). Two studies used messaging services,^55^^,^^61^ and 3 used software applications (1 online and 2 smartphone).^45^^,^^54^^,^^58^ One study aimed to facilitate EBP through the implementation of a structured model of care for patients with OA, delivered by physiotherapists and GPs.^56^

Four of the intervention studies were randomized controlled trials that tested the efficacy of interventions to enhance the effectiveness of prescribed exercises for persons with knee OA^54^^,^^55^^,^^57^ and upper- or lower-limb musculoskeletal complaints.^58^ Interventions comprised applications (smartphone and online),^54^^,^^58^ telephone-based coaching,^57^ and messaging services.^55^ Final intervention follow-up time frames varied in these studies from 4 weeks^58^ to 18 months.^55^ Each of these studies reported improvements in exercise program adherence among intervention groups (compared with controls) up to 6 months’ follow-up. One study^57^ reported adherence outcomes beyond 6 months (ie, at 12 and 18 months), and no difference was observed between the intervention group and the control group. Functional outcomes were also reported in each of these 4 studies. Functional outcomes favoring the intervention group over the control group were observed in only one of these studies, at 4 weeks’ follow-up.^58^ Pain outcomes were also available for 3 of these randomized controlled trials.^54^^,^^55^^,^^57^ Pain outcomes favored intervention groups at 6 weeks’ follow-up in one study,^54^ though no difference was observed between intervention groups and control groups when measured at 6 months,^55^^,^^57^ 12 months, and 18 months.^57^

Of the remaining 6 intervention studies, one was a proof-of-concept study of technology-enabled counseling,^60^ one was a feasibility study of technology-enabled counseling,^59^ and one was a pilot study of a reminder system via multimedia messaging service.^61^ Each of these studies (aiming to enhance exercise adherence) reported positive outcomes relating to the interventions in terms of preliminary efficacy and feasibility. One study (*n *=* *6) explored experiences of patients with hip and knee OA in using a smartphone application through a participatory study design.^45^ This study found that the absence of physiotherapist observation, input, and encouragement was a barrier, whereas competition and exercising in a group were enablers. One pilot study^61^ comprising 14 individuals with knee OA tested the effectiveness of a multimedia message reminder system (in addition to 6 once-weekly group exercise sessions), compared with a control group who completed the same group exercise program. Preliminary efficacy was assessed through a range of functional, adherence, and pain-related questionnaires. Marginal improvements in adherence and function were demonstrated but were not statistically significant. Two studies investigated integrated care models, one aiming to facilitate exercise adherence of individuals with knee OA^49^ and the other to improve the quality of care delivered to persons with hip and knee OA.^56^ The former comprised a structured exercise program with five 30-minute consultations over a 6-month period, with up to 12 telephone coaching sessions. Patients in this study valued personalized care and genuine interest and attention, and they experienced a sense of accountability to their physiotherapist (and telephone coach). The second study of persons with hip and knee OA^56^ comprised a randomized controlled trial of a structured treatment program that, for the intervention group (*n *=* *284), included a GP consultation and one exercise and education program with a physiotherapist. Both the GP and physiotherapist had received training on OA clinical guidelines. This was followed by up to 12 weeks of twice-weekly group-based exercise led by a physiotherapist. The control group (*n *=* *109) comprised usual care (eg, medication, referral to physiotherapy). Patients in the intervention group reported experiencing higher quality (as measured by the OsteoArthritis Quality Indicator questionnaire out of 100: mean difference = 18.9, 95% CI 12.7–25.1; *P* < .001) than did those in the control group.

### Risk-of-bias assessment of qualitative studies and randomized controlled trials

The risk-of-bias assessment found that 11 of the 14 qualitative studies included did not provide a statement about the researcher’s cultural or theoretical position, whereas 13 did not report on the potential influence of the researcher on the respective study (Supplemental Table S3). Of the 8 randomized controlled trials, blinding of the individuals delivering treatment (to the assignment of treatment) was applicable to 5 of them. None of these 5 studies reported doing this (Supplemental Table S4). Blinding of outcome assessors was applicable to 7 studies. It was unclear from 3 of these studies whether or not this was done; one study did not do this (Supplemental Table S4).

## Discussion

This review aimed to explore patient-related barriers to and enablers of, as well as interventions to enhance, the implementation of HVP for chronic pain. Our findings demonstrate that patients with chronic pain experience barriers to and enablers of engaging with HVP, which relate to multiple behavior change domains of the TDF. Barriers include cost and the patient’s perception of treatment efficacy. Furthermore, barriers and enablers relate to the patient’s beliefs (ie, of consequences and their own capability). Intervention studies predominantly focused on enhancing exercise adherence, for which efficacy was demonstrated.

There has been a significant amount of research on barriers to the implementation of HVP (and EBP) experienced by physiotherapists.^69^^,^^70^ Examples of barriers include workload and time pressure,^69^^,^^70^ knowledge and skills,^70^ and funding structures.^70^^,^^71^ Our review considers barriers from the perspective of patients, and similarly, there are multiple factors influencing their engagement with HVP. Perceived treatment efficacy is a notable barrier arising from the present review, given that patient outcomes underpin HVP.^16^ Previous research has evidenced improvements in perceived treatment efficacy through a multidisciplinary approach, inclusive of pain science education, psychologically informed care, and exercise-based physiotherapy management.^72^ Therapeutic alliance (ie, where the relationship between patient and clinician is characterized by empathy, rapport, collaboration, and trust and is nonjudgmental) is central to the delivery of such care,^73^ which was also identified herein as a bidirectional influence on patient engagement. This is consistent with previous research, which provides evidence that a strong therapeutic alliance can contribute to active engagement from patients^74^ and pain-related outcomes.^75^

Cost also emerged from our results as a barrier to accessing HVP. Given that cost-effectiveness is a key component of HVP,^16^ this is a significant finding. Hence, the value proposition of HVP treatments needs to be evident to patients to maximize their engagement. A patient’s perception of health care service value is also grounded in trust.^76^^,^^77^ This again highlights the importance of the therapeutic alliance in terms of cultivating a perception of value (and hence, engagement) among patients with chronic pain.^78^

The importance of challenging unhelpful beliefs held by patients with chronic pain was highlighted by the review. Identified themes, including fear of movement and beliefs about the influence of nonmodifiable factors (such as “degeneration of joints”), indicate a need to incorporate approaches to address these. Behavior change principles have been integrated into 4 of the randomized controlled trials reviewed herein, to good effect in terms of improving exercise adherence.^54^^,^^55^^,^^57^^,^^58^ However, a recent broader review of mobile applications to facilitate exercise adherence^79^ found limited integration of behavior change principles and mixed results in terms of improving adherence outcomes. Greater use of these principles in future interventions was suggested, which again might help to challenge unhelpful beliefs held by patients. In a previous study of physiotherapists, 60% reported that patient expectations of low-value treatments precluded implementation of higher-value treatments and that providing treatments counter to these expectations could adversely influence the therapeutic alliance.^71^ Although treatment expectations were reported as a barrier in one of our included studies,^50^ this was not a dominant theme, and this contrasts with these earlier findings.^71^ This is an important finding that could influence the clinical decision-making of physiotherapists—suggesting that (in the presence of a strong therapeutic alliance) patients are amenable to HVP.

With regard to enablers of HVP, a significant amount of research has again explored these from the perspective of physiotherapists.^69^^,^^70^ Enablers include professional mentorship,^71^ support networks,^71^ and funding structures that support implementation of HVP.^70^ Although the literature reflects a focus on enabling physiotherapists to make clinical decisions congruent with HVP, our findings indicate that the process of clinical decision-making needs to be in collaboration with patients, incorporating their perspectives. Patients value rapport with and trust in their physiotherapist, as described herein, which seems an important conduit through which other barriers could be mitigated and the success of interventions optimized.

Exercise adherence was most commonly investigated among the intervention studies included in this review, reflecting the weight of evidence for exercise as an effective component of HVP for chronic pain.^20^^,^^22^^,^^80^ Our findings suggest that technology-based interventions are an encouraging approach to enhancing implementation of HVP, in terms of demonstrating efficacy for increasing exercise adherence. Among patients with hip and knee OA, this has previously been shown to improve pain, functional, and perceived efficacy outcomes.^81^ However, the 4 randomized controlled trials of interventions reviewed^54^^,^^55^^,^^57^^,^^58^ demonstrate that improved adherence among intervention groups does not necessarily translate into improved pain and functional outcomes. Lack of effectiveness might reflect the challenges of implementing the optimal type and dosage of self-managed exercise programs.^82^ Aside from exercise adherence, the breadth and nature of the barriers reported herein, against the backdrop of the reviewed interventions, suggest scope to explore novel approaches to overcome them.

Although exploring terminology differences within the literature was not an aim of this review, we have observed the use of different terms relating to treatments that could be considered EBP or HVP. Examples include *high-value*,^41^*evidence-based*,^43^ and *high-quality*.^43^^,^^53^ This might be because EBP is a component of HVP or because HVP recommendations were introduced in Australia only in 2015.^13^ This observation might also indicate that the term *HVP* has not yet infiltrated the vernacular of physiotherapists and researchers alike. This seems consistent with recent work, which posits that recommendations seeking to foster HVP have lacked industry-based impact.^19^

### Strengths and limitations

Studies pertaining to hip or knee OA and CLBP are highly represented compared with other chronic pain conditions, and hence, our findings need to be considered in this context. Furthermore, having included studies of different chronic pain conditions from various settings could have influenced the barriers and enablers reported.

The risk-of-bias assessment demonstrated that the majority of the qualitative studies did not provide a statement on the researcher’s cultural or theoretical position or on the potential influence of the researcher on the study itself, which might have impacted the interpretation and presentation of findings. Furthermore, with regard to the randomized controlled trials, blinding of the individuals delivering treatment and outcome assessors was not done or reported in several studies, which could have introduced bias.

Our review of 4 randomized controlled trials predominantly comprised individuals with knee OA, and hence, the most reliable evidence we have reported on pertains mainly to this population. Notably, only one study involved patients with CNP, which, given that CNP is a cause of significant individual and societal burden,^83^ indicates a need for further research into this area. Although some chronic pain conditions share common influences on patient engagement with HVP, as shown here, there appears to be a need to better understand this in relation to patients experiencing CNP because of the related comorbidities, such as dizziness,^84^ migraine,^85^ and whiplash-associated disorders.^86^

Studies focusing on exercise adherence are also highly represented in this review, which, given the weight of evidence supporting exercise therapy for management of chronic pain,^20–22^ is a strength. Furthermore, the use of the TDF in our framework synthesis approach is also a strength of this review and provides a construct from which influences on patient engagement with HVP can be understood. This also provides a reference point for future development of interventions seeking to enhance implementation of HVP. Another strength of our review is the focus on HVP. This aligns with the strategic direction of industry stakeholders, such as the Australian Physiotherapy Association^18^ and American Physical Therapy Association,^87^ and hence, this review contributes to a deeper understanding of this concept in the context of patient-related experiences.

## Conclusion

This review shows that patients with chronic pain experience barriers to engaging with HVP, which include the nature of their interaction with their physiotherapist, the perceived efficacy of treatment, and cost. Furthermore, consideration needs to be given to patients’ beliefs in order to optimize their engagement with HVP. Patient-related interventions have predominantly aimed to increase adherence to exercise through the use of technology, and they have demonstrated moderate effectiveness. Our findings suggest that interventions seeking to enhance implementation of HVP need to consider the multifactorial barriers experienced by patients with chronic pain conditions. Doing so could enhance patient engagement and optimize their treatment outcomes.

## Supplementary Material

pnad134_Supplementary_Data
