# Supplementary material for: Patient-related barriers and enablers to the implementation of high-value physiotherapy for chronic pain: a systematic review
Source: Pain Med. 2023 Sep 28;25(2):104–15. doi: 10.1093/pm/pnad134 (PMC10833081; doi:10.1093/pm/pnad134)
Supplement: pnad134_Supplementary_Data [file pnad134_supplementary_data.zip › pnad134_Supplementary_Data/Dickson et al. 2023_POST ACCEPTANCE_SUPPLEMENTARY TABLE 1.docx]

**Table S1. Logic grid for systematic search and related synonyms**

| Title keyword abstract | "Physical therapist" OR  Rehabilitation OR  Physio* OR "Physical Therapists" OR "Physical Therapy" OR kinesiotherap* | (((Chronic OR "Long-term" OR "long term" OR dysfunctional OR persist* OR longstanding) AND pain*) OR "reflex sympathetic dystrophy" OR fibromyalgia OR "polymyalgia rheumatica" OR osteoarthrit* OR arthrit* OR "chronic regional pain syndrome" OR musculoskeletal OR "musculo-skeletal" OR "non-cancer" OR "chronic primary pain" OR widespread) | Barrier* OR enabl* OR facilitat* | "Evidence-based practice" OR "evidence based practice" OR  EBP OR "best practice" OR "clinical guidelines"  OR "quality improvement" OR "high value care" OR "high-value care" OR "low value care" OR "low-value care" OR "research-based" OR "research based" OR "gold standard care" OR "gold-standard care" |
| --- | --- | --- | --- | --- |
| Ovid Medline^1^ | "Physical Therapists" | "Chronic pain" |  | "Evidence based practice" |
| Embase^2^ | Physiotherapist | "Chronic pain" |  | "Evidence based medicine" |
| CINAHL^3^ | Physical Therapists  Physical Therapist Attitudes | "Chronic pain" |  | "Physical therapy practice, evidence based" |

**CP:** Chronic musculoskeletal pain; **EBP:** Evidence-based practice; **^1^** Ovid Medline: Medical Subject Headings

(MeSH terms); **^2^** Embase subject headings (Emtree); **^3^** CINAHL subject headings.
