# Supplementary material for: Patient-related barriers and enablers to the implementation of high-value physiotherapy for chronic pain: a systematic review
Source: Pain Med. 2023 Sep 28;25(2):104–15. doi: 10.1093/pm/pnad134 (PMC10833081; doi:10.1093/pm/pnad134)
Supplement: pnad134_Supplementary_Data [file pnad134_supplementary_data.zip › pnad134_Supplementary_Data/Dickson et al. 2023_POST ACCEPTANCE_SUPPLEMENTARY TABLE 2.docx]

**Table S2. Complete search strategy for Medline.**

| **Search ^1^** | **Syntax** |
| --- | --- |
| **1** | Exp Physical Therapists/ OR ("Physical therapist" OR Rehabilitation OR Physio* OR "Physical Therapists" OR "Physical Therapy" OR kinesiotherap*).ti,ab |
| **2** | Exp Chronic pain/ OR (((Chronic OR Long-term OR "long term" OR dysfunctional OR persist* OR longstanding) AND pain*) OR "reflex sympathetic dystrophy" OR fibromyalgia OR "polymyalgia rheumatica" OR osteoarthrit* OR arthrit* OR "chronic regional pain syndrome" OR musculoskeletal OR musculo-skeletal OR non-cancer OR "chronic primary pain" OR widespread).ti,ab |
| **3** | (Barrier* OR enabl* OR facilitat*).ti,ab |
| **4** | Exp Evidence based practice/ OR ("Evidence-based practice" OR "evidence based practice" OR EBP OR best practice OR "clinical guidelines" OR "quality improvement" OR "high value care" OR "high-value care" OR "low value care" OR "low-value care" OR research-based OR "research based" OR "gold standard care" OR "gold-standard care").ti,ab |

^1^ Searches 1-4 combined with “AND”.
