# Supplementary material for: Patient-related barriers and enablers to the implementation of high-value physiotherapy for chronic pain: a systematic review
Source: Pain Med. 2023 Sep 28;25(2):104–15. doi: 10.1093/pm/pnad134 (PMC10833081; doi:10.1093/pm/pnad134)
Supplement: pnad134_Supplementary_Data [file pnad134_supplementary_data.zip › pnad134_Supplementary_Data/Dickson et al. 2023_POST ACCEPTANCE_SUPPLEMENTARY TABLE 3.docx]

| **Table S3: Joanna Briggs Institute Critical Appraisal Checklist for Qualitative Research.** | | | | | | | | | | | | | | | |
| --- | --- | --- | --- | --- | --- | --- | --- | --- | --- | --- | --- | --- | --- | --- | --- |
|  |  | **Boyle et al. 2022** | **Joyce et al. 2022** | **Teo et al. 2021** | **Garrett et al. 2021** | **Meerhoff et al. 2021** | **Smith et al. 2019** | **Saner et al. 2018** | **Danbjorg et al. 2018** | **Palazzo et al. 2016** | **Withall et al. 2016** | **Escolar-Reina et al. 2010** | **Poitras et al. 2010** | **Campbell et al. 2001** | **Hinman et al. 2016** |
| **1** | Is there congruity between the stated philosophical perspective and the research methodology? | Y | Y | Y | Y | Y | Y | Y | Y | Y | Y | Y | Y | Y | Y |
| **2** | Is there congruity between the research methodology and the research question or objectives? | Y | Y | Y | Y | Y | Y | Y | Y | Y | Y | Y | Y | Y | Y |
| **3** | Is there congruity between the research methodology and the methods used to collect data? | Y | Y | Y | Y | Y | Y | Y | Y | Y | Y | Y | Y | Y | Y |
| **4** | Is there congruity between the research methodology and the representation and analysis of data? | Y | Y | Y | Y | Y | Y | Y | Y | Y | Y | Y | Y | Y | Y |
| **5** | Is there congruity between the research methodology and the interpretation of results? | Y | Y | Y | Y | Y | Y | Y | Y | Y | Y | Y | Y | Y | Y |
| **6** | Is there a statement locating the researcher culturally or theoretically? | N | N | N | N | N | Y | N | Y | N | Y | N | N | N | N |
| **7** | Is the influence of the researcher on the research, and vice- versa, addressed? | N | N | N | N | N | Y | N | N | N | N | N | N | N | N |
| **8** | Are participants, and their voices, adequately represented? | Y | Y | Y | Y | Y | Y | Y | Y | Y | Y | Y | Y | Y | Y |
| **9** | Is the research ethical according to current criteria or, for recent studies, and is there evidence of ethical approval by an appropriate body? | Y | Y | Y | Y | Y | Y | Y | Y | Y | Y | Y | Y | U | Y |
| **10** | Do the conclusions drawn in the research report flow from the analysis, or interpretation, of the data? | Y | Y | Y | Y | Y | Y | Y | Y | Y | Y | Y | Y | Y | Y |
|  | |  |  |  |  |  |  |  |  |  |  |  |  |  |  |
