# Supplementary material for: Patient-related barriers and enablers to the implementation of high-value physiotherapy for chronic pain: a systematic review
Source: Pain Med. 2023 Sep 28;25(2):104–15. doi: 10.1093/pm/pnad134 (PMC10833081; doi:10.1093/pm/pnad134)
Supplement: pnad134_Supplementary_Data [file pnad134_supplementary_data.zip › pnad134_Supplementary_Data/Dickson et al. 2023_POST ACCEPTANCE_SUPPLEMENTARY TABLE 4.docx]

| **Table S4: Joanna Briggs Institute Critical Appraisal Checklist for Randomised-Controlled Trials.** | | | | | | | | | |
| --- | --- | --- | --- | --- | --- | --- | --- | --- | --- |
|  |  | **Alasfour et al. 2022** | **Bennell et al. 2020** | **Osteras et al. 2019** | **Bennell et al. 2017** | **Lambert et al. 2017** | **Li et al. 2018** | **Li et al. 2017** | **Dar et al. 2014** |
| **1** | Was the randomization used for assignment of participants to treatment groups? | Y | Y | Y | Y | Y | Y | Y | Y |
| **2** | Was allocation to treatment groups concealed? | Y | Y | Y | Y | Y | Y | Y | U |
| **3** | Were treatment groups similar at the baseline? | Y | Y | NA | Y | Y | Y | Y | N |
| **4** | Were the participants blind to treatment assignment? | N | Y | NA | N | N | Y | Y | N |
| **5** | Were those delivering the treatment blind to treatment assignment? | N | N | N | N | NA | NA | NA | N |
| **6** | Were treatment groups treated identically other than the intervention of interest? | Y | Y | Y | Y | Y | Y | Y | Y |
| **7** | Were outcome assessors blind to treatment assignment? | N | Y | NA | U | Y | Y | U | U |
| **8** | Were outcomes measured in the same way for treatment groups? | Y | Y | Y | Y | Y | Y | Y | Y |
| **9** | Were outcomes measured in a reliable way | U | Y | Y | Y | Y | Y | Y | Y |
| **10** | Was follow up complete and if not, were differences between groups in terms of their follow up adequately described and analysed? | Y | Y | Y | Y | Y | N | Y | Y |
| **11** | Were participants analysed in the groups to which they were randomized? | Y | Y | Y | Y | Y | Y | Y | Y |
| **12** | Was appropriate statistical analysis used? | Y | Y | Y | Y | Y | Y | Y | Y |
| **13** | Was the trial design appropriate and any deviations from the standard RCT design (individual randomization, parallel groups) accounted for in the conduct and analysis of the trial? | Y | Y | Y | Y | Y | Y | Y | Y |
